# Heat Transfer in MHD Mixed Convection Flow of a Ferrofluid along a Vertical Channel

**DOI:** 10.1371/journal.pone.0141213

**Published:** 2015-11-09

**Authors:** Aaiza Gul, Ilyas Khan, Sharidan Shafie, Asma Khalid, Arshad Khan

**Affiliations:** 1 Department of Mathematical Sciences, Faculty of Science, Universiti Teknologi Malaysia, UTM Skudai, Johor, Malaysia; 2 College of Engineering, Majmaah University, Majmaah, Saudi Arabia; 3 Sardar Bahadur Khan Women’s University, Quetta, Pakistan; Abdul Wali Khan university Mardan Pakistan, PAKISTAN

## Abstract

This study investigated heat transfer in magnetohydrodynamic (MHD) mixed convection flow of ferrofluid along a vertical channel. The channel with non-uniform wall temperatures was taken in a vertical direction with transverse magnetic field. Water with nanoparticles of magnetite (*Fe*_3_ *O*_4_) was selected as a conventional base fluid. In addition, non-magnetic (*Al*_2_ *O*_3_) aluminium oxide nanoparticles were also used. Comparison between magnetic and magnetite nanoparticles were also conducted. Fluid motion was originated due to buoyancy force together with applied pressure gradient. The problem was modelled in terms of partial differential equations with physical boundary conditions. Analytical solutions were obtained for velocity and temperature. Graphical results were plotted and discussed. It was found that temperature and velocity of ferrofluids depend strongly on viscosity and thermal conductivity together with magnetic field. The results of the present study when compared concurred with published work.

## Introduction

The idea of using small-sized solid particles inside fluids to increase their thermal conductivity was initially initiated by Maxwell [1]. This idea was based on suspension of micro-sized or milli-sized solid particles inside fluids. Subsequently, however it was realized that large sized particles in the milli- scale or even micro-sized particles causes several technical problems. For example, (i) faster settling time, (ii) clogging micro-channels of devices, (iii) abrasion of surfaces (iv) erosion of pipelines and (v) increasing drop in pressure [2]. Based on these problems, Choi [3] came up with the idea of improving thermal conductivity using nano-sized particles. More specifically, Choi experimentally verified that the addition of nanoparticles in conventional based fluids enhances the thermal conductivity and the mixture which was composed of base fluid with suspended nanoparticles named as nanofluid. Besides, higher thermal conductivity, the addition of nano-sized particles over micro-sized particles to conventional base fluid was preferred due to several valid scientific reasons such as (i) longer suspension time (more stable), larger surface area/volume ration (1000 times larger), (iii) lower erosion and clogging, (iv) lower demand for pumping power (v) reduction in inventory of heat transfer fluid, and (vi) significant energy saving [4].

Nanofluids have numerous applications in nano-scale flow. In this type of flow the molecular structure of the fluid and surfaces, andinteraction between them at the atomistic length scale play a key role and hence the molecular dynamics (MD) method emerges as a viable approach for investigation of the flow in such scale. Examples of nano-scale flows include bubbfil spinning, bubble electrospinning, blown bubble spinning, membrane spinning processes used for nanofiber fabrication. Bubbfil spinning is a nanoscale flow process in which polymer/melts, bubbles/membranes are used for fabrication of nanofibers by using electrostatic force, flowing air or mechanical force (e.g. centrifugal force) to overcome the surface tension of bubble[5]. Chen et al. [6]studied bubbfil spinning process for mass-production of nanofibers. He et al. [7] analyzed various characteristics of Nanoscale flow with some interesting results.

On one hand, some commonly used fluids such as water, ethylene glycol, and mineral oils are found to have poor thermal characteristics when compared with metals, non-metals and their oxides. On the other hand, it is noticed that the flow analysis of nanofluids with the interaction of magnetic field have increased enormously. Perhaps this could be due to the numerous industrial and engineering applications of nanofluids. There are three categories which describe how a material is equivalently affected by a magnetic field. There are three types of magnetic materials namely: (i) Diamagnetism: materials such as copper, lead, quartz, water, acetone, and carbon dioxide are diamagnetic. These materials are very weakly affected by magnetic fields, (ii) Paramagnetism: materials such as sodium, oxygen, iron oxide (*FeO or Fe*_3_ *O*_4_), and platinum are paramagnetic. They are affected somewhat more strongly than diamagnetic materials, and become polarized parallel to a magnetic field (iii) Ferromagnetic: ferromagnetic materials include gadolinium, iron, iron oxide (magnetite) (*Fe*_3_ *O*_4_), and nickel, cobalt ferrite (*CoFe*_3_ *O*_4_) and manganese bismuth (*MnBi*). These materials are very strongly affected by magnetic fields. In addition, they become strongly polarized in the direction of the magnetic field and retain their polarization state after the magnetic field is removed. Together, all these three types of materials are called magnets or ferromagnetic materials [8]. Ram and Kumar [9] noticed that temperature of the ferrofluids decreases with the increase of viscosity radiation parameter. The magnetohydrodynamic stagnation point flow of a nanofluid over a stretching/shrinking sheet with suction was studied by Mansur et al. [10]. It was found that temperature of the ferrofluids decrease with the increase of viscosity radiation parameter. Colla et al. [11] investigated Water-Based *Fe*_3_ *O*_4_ nanofluid characterization: thermal conductivity and viscosity measurements and correlation. Colla and Ram have found the same results of viscosity variation with the temperature. Abareshi et al. [12] studied fabrication, characterization and measurement of thermal conductivity of *Fe*_3_ *O*_4_ nanofluids. Borglin et al. [13] studied experimentally the flow of a ferrofluid in porous media.

Amongst these three types, ferromagnetic materials produce a strong magnetic field. Therefore, in the present work, we had chosen nanoparticles of magnetite (*Fe*_3_ *O*_4_), being the most commonly used magnetic material and water was chosen as a conventional base fluid. The resulting fluid was called ferrofluid also known as magnetic fluid. More specifically, ferrofluids are colloidal suspensions of small magnetic particles in a carrier liquid. Some important uses of ferrofluids are found in mechanical damping in loudspeakers and in heat exchangers.

Based on the importance of ferromagnetic materials, Qasim et al. [14] examined MHD flow with slip condition in the presence of heat transfer in ferrofluid over a stretched cylinder with given heat flux. They used water as conventional base fluid and added magnetite (*Fe*_3_ *O*_4_) nanoparticles. For the sake of comparison they also added non-magnetic (*Al*_2_ *O*_3_) nanoparticles to the base fluid. Khan et al. [15] tackled a stagnation point problem of ferrofluid along a stretching sheet with viscous dissipation and heat transfer. They considered ferroparticles of three types: magnetite (*Fe*_3_ *O*_4_), cobalt ferrite (*CoFe*_3_ *O*_4_), and *Mn*−*Zn* ferrite (*Mn*−*ZnFe*_3_ *O*_4_). However, they selected two types of base fluid, water and kerosene and found some interesting results for these two types of base fluids after using implicit finite-difference method with quasi-linearization technique as the solution to a resultant problem. By using ferrofluid, Sheikholeslami and Ganji [16] investigated heat transfer with thermal radiation inside an enclosure of semi annulus in the presence of a magnetic source. Idress et al. [17] studied application of the optimal homotopy asymptotic method for the solution of the Korteweg-de Vries equation. Ellahi et al. [18] studied series solutions of non-Newtonian nanofluids with Reynolds' model and Vogel's model by means of the homotopy analysis method. Herisanu and Vasile [19] investigated that optimal homotopy perturbation method for a non-conservative dynamical system of a rotating electrical machine. Several other studies were conducted in the last few years, on nanofluids by taking different types of convectional base fluids with different nanoparticles, see for example [20–29] and the related references therein.

The above literature review revealed that no study has yet to be conducted on heat transfer in mixed convection flow of ferrofluid inside a vertical channel in the presence of a magnetic field. Also, the interaction of magnetic field with magnetite nanoparticles in a mixed convection flow, presents an interesting fluid dynamics problem [30]. Therefore, this study specifically investigates the behavior of water suspension which contains nanoparticles of magnetite (*Fe*_3_ *O*_4_) making as ferrofluid together with heat transfer analysis due to mixed convection. In this problem non-magnetic (*Al*_2_ *O*_3_) nanoparticles were also used and a comparison between magnetic and non-magnetic nanoparticles is conducted. Analytical solutions are obtained, plotted graphically and discussed.

## Problem Formulation and Solution

In formulating the problem, this study considered water-based nanofluid containing magnetite (Fe3O4) nanoparticles. Pressure gradient of oscillatory type was applied in the flow direction. Free convection in the presence of radiation effect was also considered. Mixed convection flow was induced inside a vertical channel of width *d* with constant temperature. Fluid was electrically conducted due to magnetic field **B**_0_ of strength *B*_0_ applied in a transverse direction to the flow. Magnetic Reynolds number chosen was small and induced magnetic field was neglected. External electric field and electric field due to polarization were taken as zero. Top boundary of the channel was maintained at constant temperature *T*_*w*_ while the bottom boundary had a uniform temperature *T*_0_. Channel was taken along the *x* − axis and *y* − axis was taken as normal to the flow direction.

Based on the approximation of Boussinesq and taking into consideration the above assumptions, the governing equations of momentum and energy obtained are as follows:

$$\rho_{nf}\frac{\partial v}{\partial t}=-\frac{\partial p}{\partial x}+\mu_{nf}\frac{\partial^{2}v}{\partial y^{2}}-\sigma_{nf}B_{0}^{2}v+{\left(\rho \beta \right)}_{nf}g\left(T-T_{0}\right),$$ (1)

$${\left(\rho c_{p}\right)}_{nf}\frac{\partial T}{\partial t}=k_{nf}\frac{\partial^{2}T}{\partial y^{2}}-\frac{\partial q}{\partial y}\;,$$ (2)

with boundary conditions

$$v(0,t)=0,\;T(0,t)=T_{0},$$ (3)

$$v(d,t)=0,\;T(d,t)=T_{w},$$ (4)

where *v* = *v*(*y*, *t*) denotes the fluid velocity in the *x* − direction, *T* = *T*(*y*, *t*) is the temperature of the nanofluid, *ρ*_*nf*_ is the density of the nanofluid, *μ*_*nf*_ is the dynamic viscosity of the nanofluid, *σ*_*nf*_ is the electrical conductivity of the nanofluid, *β*_*nf*_ is the volumetric coefficient of thermal expansion of the nanofluid, *g* is the acceleration due to gravity, *k*_*nf*_ is the thermal conductivity of the nanofluid, (*c*_*p*_)_*nf*_ is the specific heat of the nanofluid at constant pressure, *q* is the radiative heat flux in *x* − direction, and *d* is the width of the channel. With reference to [14,15,22,26], the relations of density, dynamic viscosity and thermal conductivity of the nanofluid with corresponding base fluid are given as:

(ρcp)nf=(1−φ)(ρcp)f+φ(ρcp)s,ρnf=(1−φ)ρf+φρS,σnf=αnf(ρcp)nf,(ρβ)nf=(1−φ)(ρβ)f+φ(ρβ)S,μnf=μf(1−φ)2.5,knfkf=(ks+2kf)−2φ(kf−ks)(ks−2kf)+φ(kf−ks), (5)

where *φ* is the volume fraction of the nanoparticles, *ρ*_*f*_ is the density of the base fluid, *ρ*_*s*_ is the density of the solid nanoparticles, *μ*_*f*_ is the dynamic viscosity of the base fluid, *k*_*f*_ and *k*_*s*_ are the thermal conductivities of the base fluid and solid nanoparticles respectively, (*c*_*p*_)_*f*_ and (*c*_*p*_)_*s*_ denote the specific heat at constant pressure corresponding to the base fluid and solid nanoparticles. The total term (*ρc*_*p*_) is termed as heat capacitance. According to Turkyilmazoglu [26], the relations in Eq 5 are restricted to nanoparticles with spherical shape. For nanoparticles of other shapes with different thermal conductivity and dynamic viscosity, Eq 5 would be modified accordingly. The corresponding ferrofluid or nanofluid thermophysical properties are given in Tables 1 and 2. These properties would be used in numerical computations of this problem [14,15,22]. However, these values would be required in numerical computations, when plotting graphs.

**Table 1:** Spherical and cylindrical shaped nanoparticles with dynamic viscosity and thermal Conductivity.

No	Shape of Nanoparticles	Dynamic Viscosity	Thermal Conductivity
1	Spherical	$\mu_{nf}=\frac{\mu_{f}}{{\left(1-\varphi \right)}^{2.5}}$	$\frac{k_{nf}}{k_{f}}=\frac{\left(k_{s}+2k_{f})-2\varphi (k_{f}-k_{s}\right)}{\left(k_{s}-2k_{f})+\varphi (k_{f}-k_{s}\right)}$
2	Spherical	*μ*_*nf*_ = *μ*_*f*_(1 + 7.3*φ* + 123*φ* ^2^)	$\frac{k_{nf}}{k_{f}}=\frac{\left(k_{s}+2k_{f})-2\varphi (k_{f}-k_{s}\right)}{\left(k_{s}-2k_{f})+\varphi (k_{f}-k_{s}\right)}$
3	Cylindrical	$\mu_{nf}=\frac{\mu_{f}}{{\left(1-\varphi \right)}^{2.5}}$	$\frac{k_{nf}}{k_{f}}=\frac{\left(k_{s}+\frac{1}{2}k_{f})-\frac{1}{2}\varphi (k_{f}-k_{s}\right)}{\left(k_{s}-\frac{1}{2}k_{f})+\varphi (k_{f}-k_{s}\right)}$
4	Cylindrical	*μ*_*nf*_ = *μ*_*f*_(1 + 7.3*φ* + 123*φ* ^2^)	$\frac{k_{nf}}{k_{f}}=\frac{\left(k_{s}+\frac{1}{2}k_{f})-\frac{1}{2}\varphi (k_{f}-k_{s}\right)}{\left(k_{s}-\frac{1}{2}k_{f})+\varphi (k_{f}-k_{s}\right)}$

**Table 2:** Thermophysical properties of base fluid (water) and nanoparticles (iron oxide and alumina oxide).

Model	*c*_*p*_(*kg* ^−1^ *K* ^−1^)	*ρ*(*kgm* ^−3^)	*k*(*Wm* ^−1^ *K* ^−1^)	*β*×10^−5^ (*K* ^−1^)
H_2_O	4179	997.1	0.613	21
Al_2_O_3_	765	3970	40	0.85
Fe_3_O_4_	670	5180	9.7	0.5

Based on Makinde and Mhone [30], the radiative heat flux is as follows:

$$-\frac{\partial q}{\partial y}=4\alpha_{0}^{2}\left(T-T_{0}\right),$$ (6)

where *α*_0_ is the mean radiation absorption coefficient. Applied pressure gradient in the flow direction was taken as −∂*p* / ∂*x* = *λε exp*(*iω*_1_ *τ*), where *λ* is constant and *ω*_1_ is the frequency of oscillation.

The following dimensionless variables are introduced:

$$\xi =\frac{x}{d},\;\eta =\frac{y}{d},\;u=\frac{v}{U_{0}},\;\tau =\frac{tU_{0}}{d},\;P=\frac{d}{\mu U_{0}}p,\;\theta =\frac{T-T_{0}}{T_{w}-T_{0}},\;\omega =\frac{d\omega_{1}}{U_{0}},\;\frac{\partial P}{\partial \xi }=\lambda \varepsilon e^{i\omega_{1}\tau },\;$$ (7)

The system of equations (1)–(4) is reduced to:

$$a_{0}\frac{\partial u}{\partial \tau }=\lambda \varepsilon e^{i\omega \tau }+\phi_{2}\frac{\partial^{2}u}{\partial \eta^{2}}-m_{0}^{2}\;u-a_{1}\;\theta ,$$ (8)

u(0,τ)=0,u(1,τ)=0,τ>0 (9)

$$b_{0}\frac{\partial \theta }{\partial \tau }=\frac{\partial^{2}\theta }{\partial \eta^{2}}+b^{2}{}_{1}\theta ,$$ (10)

θ(0,τ)=0;θ(1,τ)=0,τ>0, (11)

where

$$\varphi_{1}=\left[(1-\varphi )+\varphi \frac{\rho_{s}}{\rho_{f}}\right];\;\varphi_{2}=\left[\frac{1}{{\left(1-\varphi \right)}^{2.5}}\right],$$

$$\varphi_{3}=\left[(1-\varphi )\rho_{f}+\varphi \frac{{\left(\rho \beta \right)}_{s}}{\beta_{f}}\right];\varphi_{4}=\left[(1-\varphi )+\varphi \frac{{\left(\rho {\text{c}}_{p}\right)}_{s}}{{\left(\rho {\text{c}}_{p}\right)}_{f}}\right],$$

$$\begin{array}{l} \text{Re}=\frac{U_{0}d}{v},\;a_{0}=\varphi \text{Re},\;a_{1}=\varphi_{3}Gr,\;M^{2}=\frac{\sigma_{nf}B_{0}^{2}d^{2}}{\mu_{nf}},\;Gr=\frac{g\beta_{f}d^{2}\left(T_{w}-T_{0}\right)}{\nu_{f}U_{0}},\\ N^{2}=\frac{4d^{2}\alpha_{0}^{2}}{k_{nf}},\;Pe=\frac{{\left(\rho c_{p}\right)}_{f}}{k_{f}},\;\lambda_{n}=\frac{k_{f}}{k_{nf}},\;b_{1}=\sqrt{\frac{N^{2}}{\lambda_{n}}},\;b_{0}=\frac{Pe\varphi_{4}}{\lambda_{n}}. \end{array}$$

Here *M*, Re, *N*, *Pe*, and *Gr* denote magnetic parameter, Reynold's number, radiation parameter, Peclet number and thermal Grashof number, respectively. Other arbitrary constants were used for the sake of simplification.

In order to solve Eqs 8–11, solutions of the forms

$$u\left(\eta ,\tau \right)=\left[u_{0}\left(\eta \right)+\varepsilon exp\left(i\omega \tau \right)u_{1}\left(\eta \right)\right],$$ (12)

$$\theta \left(\eta ,\tau \right)=\left[\theta_{0}\left(\eta \right)+\varepsilon exp\left(i\omega \tau \right)\;\theta_{1}\left(\eta \right)\right].$$ (13)

Using Eqs 12 and 13 in Eqs 8–11, the following system of ordinary differential equations are obtained:

$$\frac{d^{2}u_{0}\left(\eta \right)}{d\eta^{2}}-m_{1}^{2}u_{0}\left(\eta \right)=a_{2}\theta_{0}\left(\eta \right),$$ (14)

$$u_{0}\left(0\right)=0,u_{0}\left(1\right)=0,$$ (15)

$$\frac{d^{2}u_{1}}{d\eta^{2}}-m_{2}^{2}u_{1}\left(\eta \right)=-\lambda ,$$ (16)

$$u_{1}\left(0\right)=0,u_{1}\left(1\right)=0,$$ (17)

$$\frac{d^{2}\theta_{0}\left(\eta \right)}{d\eta^{2}}+b^{2}{}_{1}\theta_{0}\left(\eta \right)=0,$$ (18)

$$\theta_{0}\left(0\right)=0,\theta_{0}\left(1\right)=1,$$ (19)

$$\frac{d^{2}\theta_{1}\left(\eta \right)}{d\eta^{2}}+m_{3}^{2}\theta_{1}\left(\eta \right)=0,$$ (20)

$$\theta_{1}\left(0\right)=0,\theta_{1}\left(1\right)=0,$$ (21)

where

$$m_{1}=\frac{m^{2}{}_{0}}{\varphi_{2}}\;,\;m_{2}=\sqrt{m_{0}^{2}+i\omega a_{0}}\;,\;m_{3}=\sqrt{b_{1}-i\omega b_{0}}\;,\;a_{2}=\frac{a_{1}}{\varphi_{2}}.$$

Solutions of Eqs 18–21 yield

$$\theta_{0}\left(\eta \right)=\frac{sinb_{1}\eta }{sinb_{1}},$$ (22)

$$\theta_{1}(\eta )=0.$$ (23)

Now Eq 13 after using Eqs 22 and 23 becomes

$$\theta (\eta ,\tau )=\theta (\eta )=\frac{sinb_{1}\eta }{sinb_{1}}$$ (24)

Incorporate Eq 24 into Eq 14, and then the solution of the resulting equation with boundary conditions (Eq 15) yields

$$u_{0}\left(\eta \right)=c_{1}sinh\left(m_{1}\right)\eta +c_{2}coshm_{1}\eta -\frac{a_{2}}{\left(b_{1}^{2}+m_{1}^{2}\right)}\frac{sinb_{1}\eta }{sinb_{1}}.$$ (25)

Similarly, Eq 16 with boundary conditions (Eq 17) results in

$$u_{1}\left(\eta \right)=c_{3}sinhm_{2}\eta +c_{4}coshm_{2}\eta +\frac{\lambda }{m_{2}^{2}\varphi_{2}}.$$ (26)

Here *c*_1_, *c*_2_, *c*_3_ and *c*_4_ are arbitrary constants given by

$$c_{1}=\frac{a_{2}+b_{1}^{2}+m_{1}^{2}}{sinhm_{1}(b_{1}^{2}+m_{1}^{2})},\;c_{2}=0,\;c_{3}=\frac{\lambda }{m_{2}^{2}\varphi_{2}}\frac{1}{sinhm_{2}}(coshm_{2}-1),{\text{c}}_{4}=-\frac{\lambda }{m_{2}^{2}\varphi_{2}}.$$

Use Eqs 25 and 26 in Eq 12,

$$\begin{array}{l} u(\eta ,\tau )=\frac{(a_{2}+b_{1}^{2}+m_{1}^{2})sinhm_{1}\eta }{sinhm_{1}(b_{1}^{2}+m_{1}^{2})}-\frac{a_{2}sinb_{1}\eta }{(b_{1}^{2}+m_{1}^{2})sinb_{1}}\\ \;+\varepsilon expi\omega \tau \left[\frac{\lambda (coshm_{2}-1)}{m_{2}^{2}\varphi_{2}sinhm_{2}}sinhm_{2}\eta -\frac{\lambda }{m_{2}^{2}\varphi_{2}}coshm_{2}\eta +\frac{\lambda }{m_{2}^{2}\varphi_{2}}\right]. \end{array}$$ (27)

### Skin-friction and Nusselt number

Skin-friction and Nusselt number are evaluated from Eqs 27 and 24 as:

cf=(a2+b12+m12)m1sinhm1(b12+m12)−a2b1(b12+m12)sinb1+εexpiωτ[λm2(coshm2−1)m22φ2sinhm2] (28)

$$Nu=\frac{b_{1}}{sinb_{1}}.$$ (29)

## Results and Discussion

This section includes graphical results with illustration. Note that in this work, spherical nanoparticles were chosen whereas dynamic viscosity and thermal conductivity of spherical nanoparticles are given in Table 1. In Table 2, thermophysical properties of base fluid (water) and nanoparticles (iron oxide and alumina oxide) are given. For the numerical computation, the corresponding ferrofluid or nanofluid thermophysical properties are used from Tables 1 and 2.

**Fig 1:**
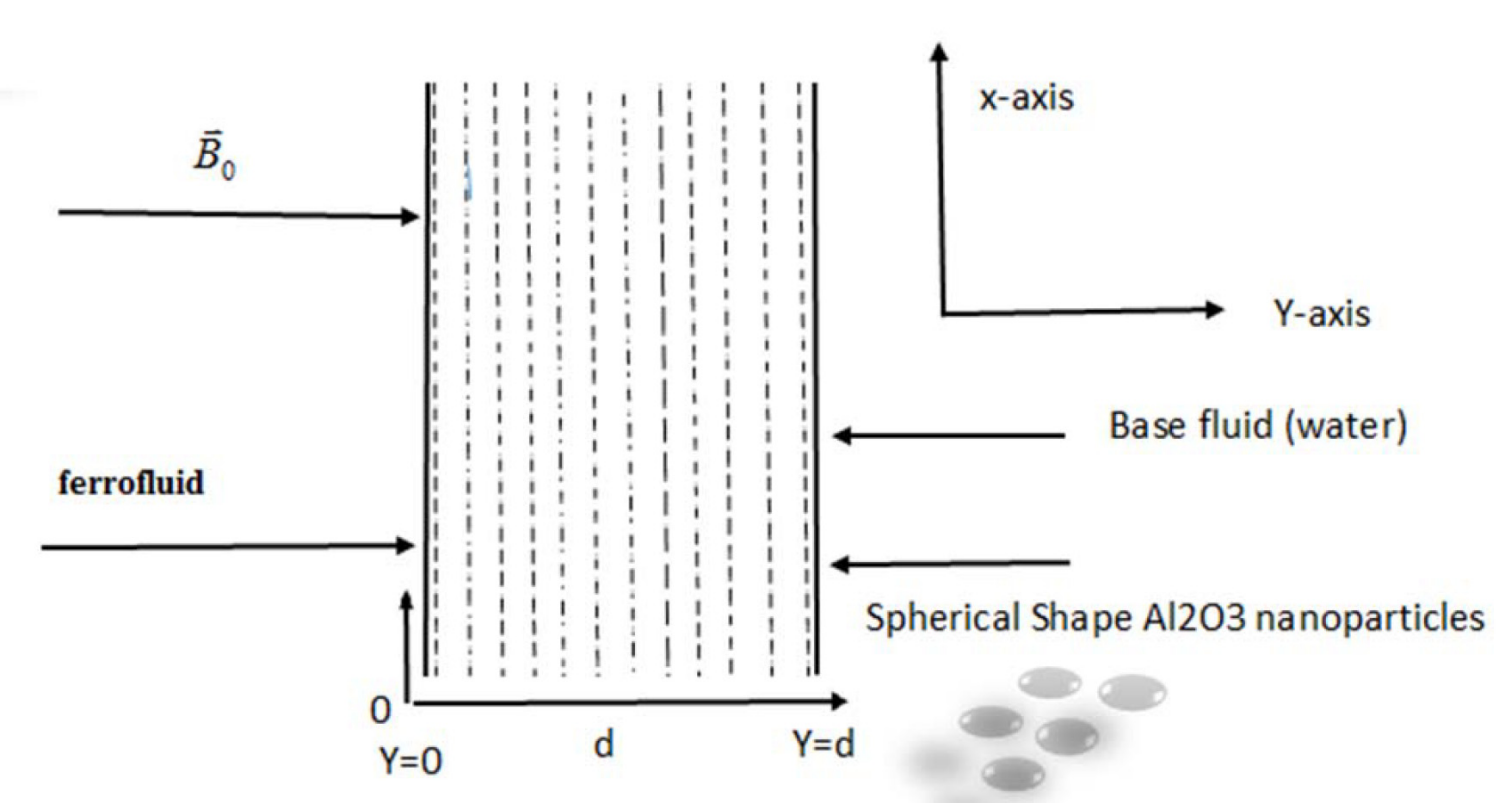
Flow geometry.

**Fig 2:**
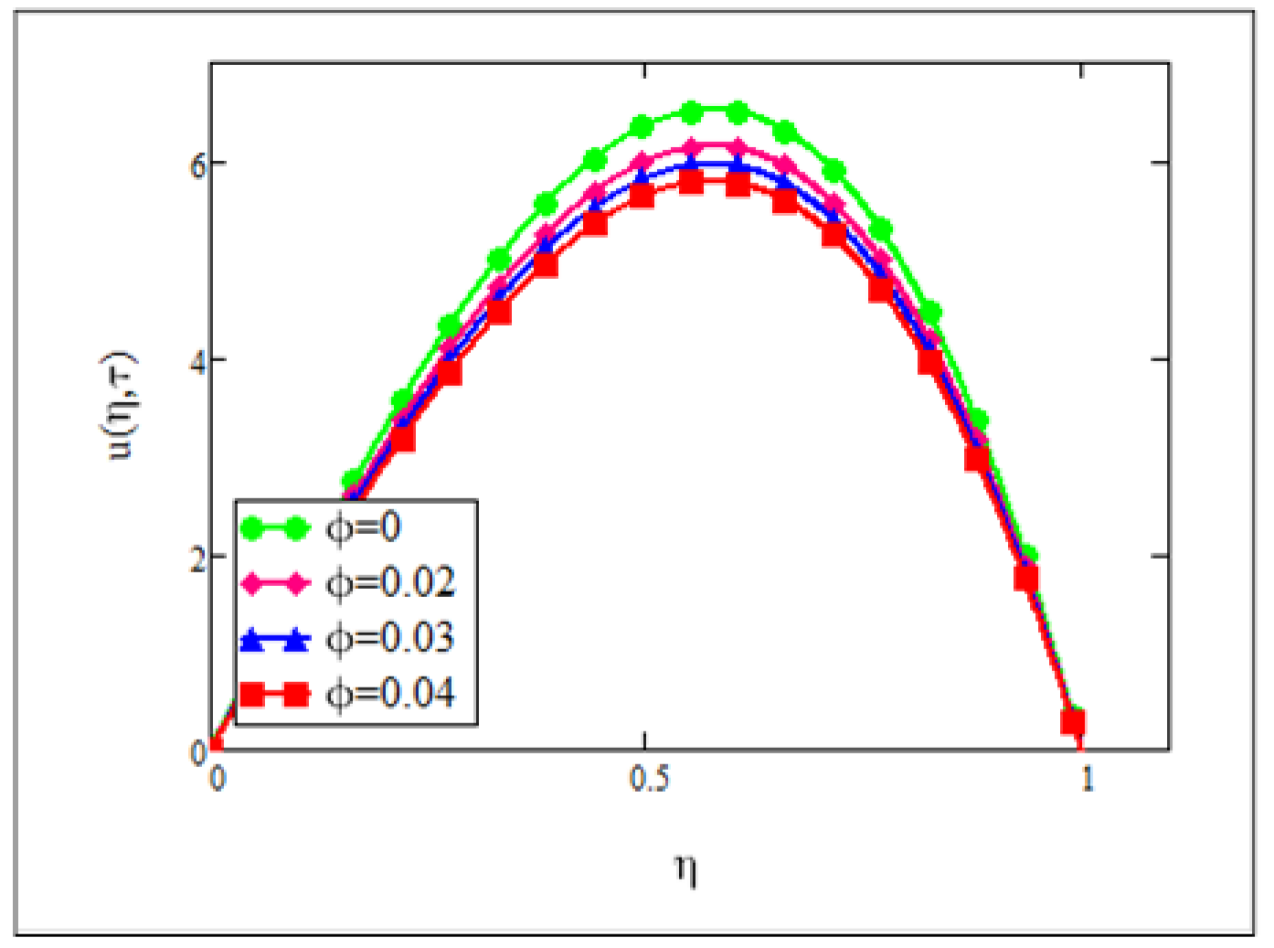
Velocity graph for *φ* = 0.0, 0.02, 0.03, 0.04 when *Gr* = 0.1, *N* = 1, Re =1, *Pe* = 1, *M* = 1, *λ* = 1, *τ* = 5 and *ω* = 0.2.

**Fig 3:**
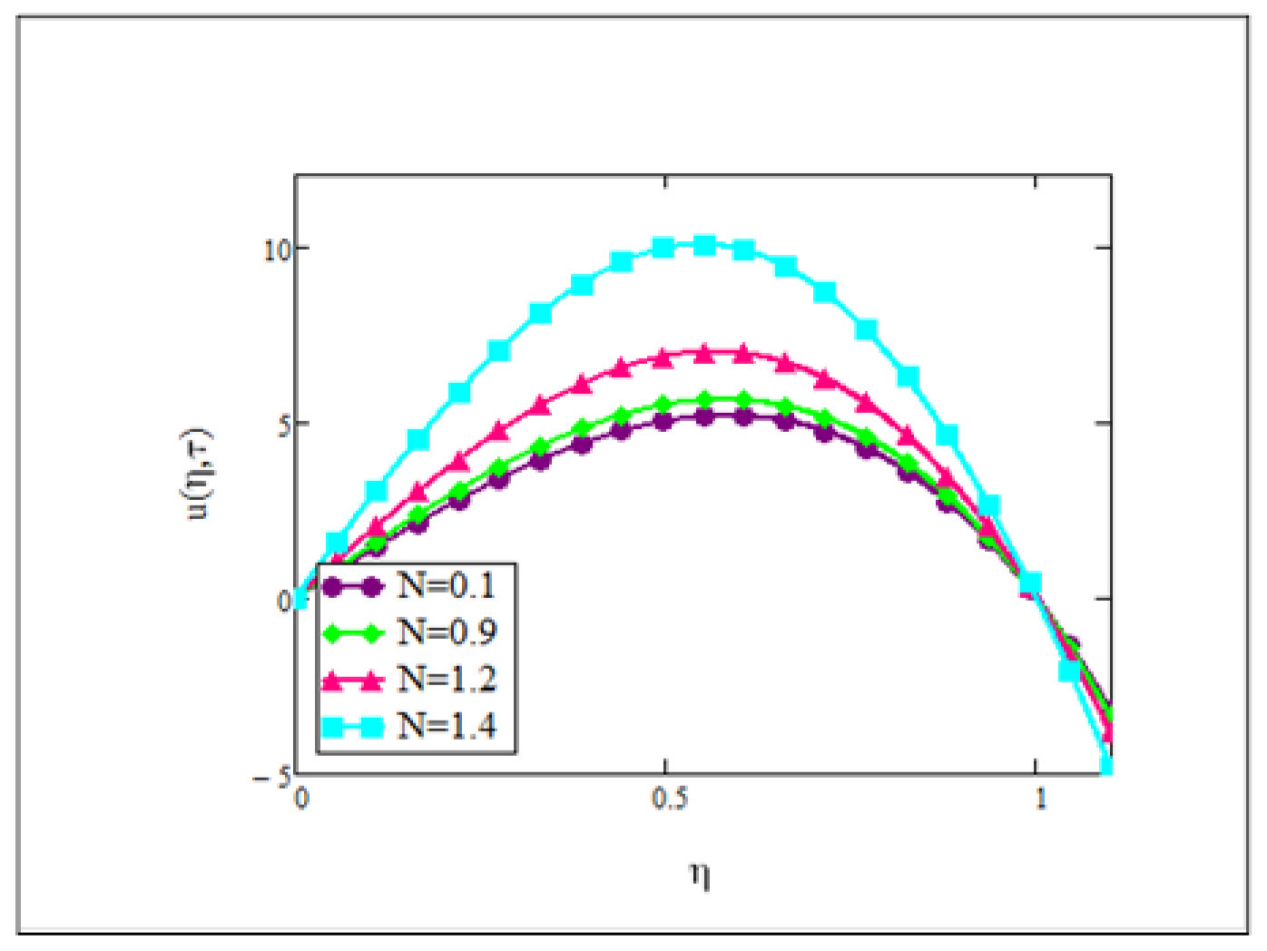
Velocity graph for *N* = 0.1, 0.9, 1.2, 1.4 when *Gr* = 0.1, *φ* = 0.04, Re = 1, *Pe* = 1, *M* = 1, *λ* = 1, *τ* = 5 and *ω* = 0.2.

**Fig 4:**
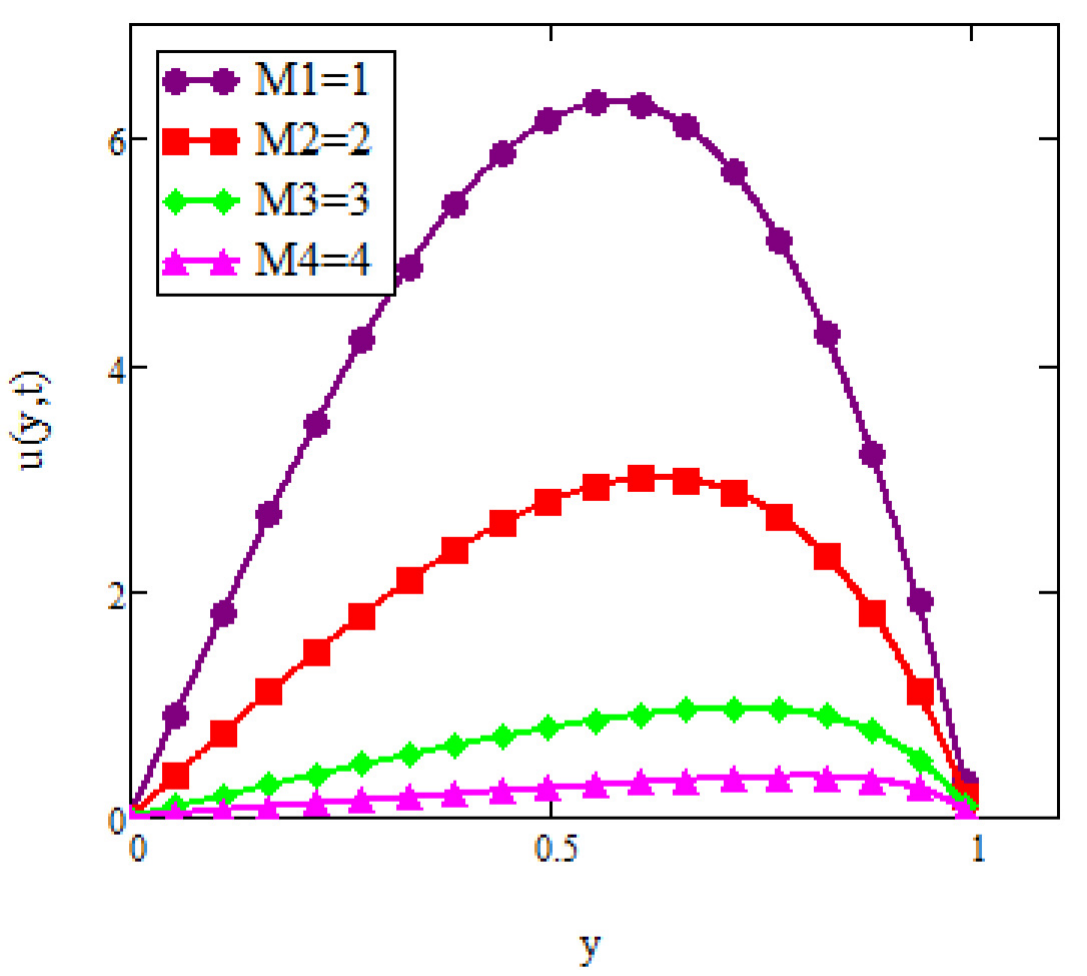
Velocity graph for *M* = 0, 2, 4, 5 when *Gr* = 0.1, *φ* = 0.04, Re = 1, *Pe* = 1, *N* = 1, *λ* = 1, *τ* = 5 and *ω* = 0.2.

**Fig 5:**
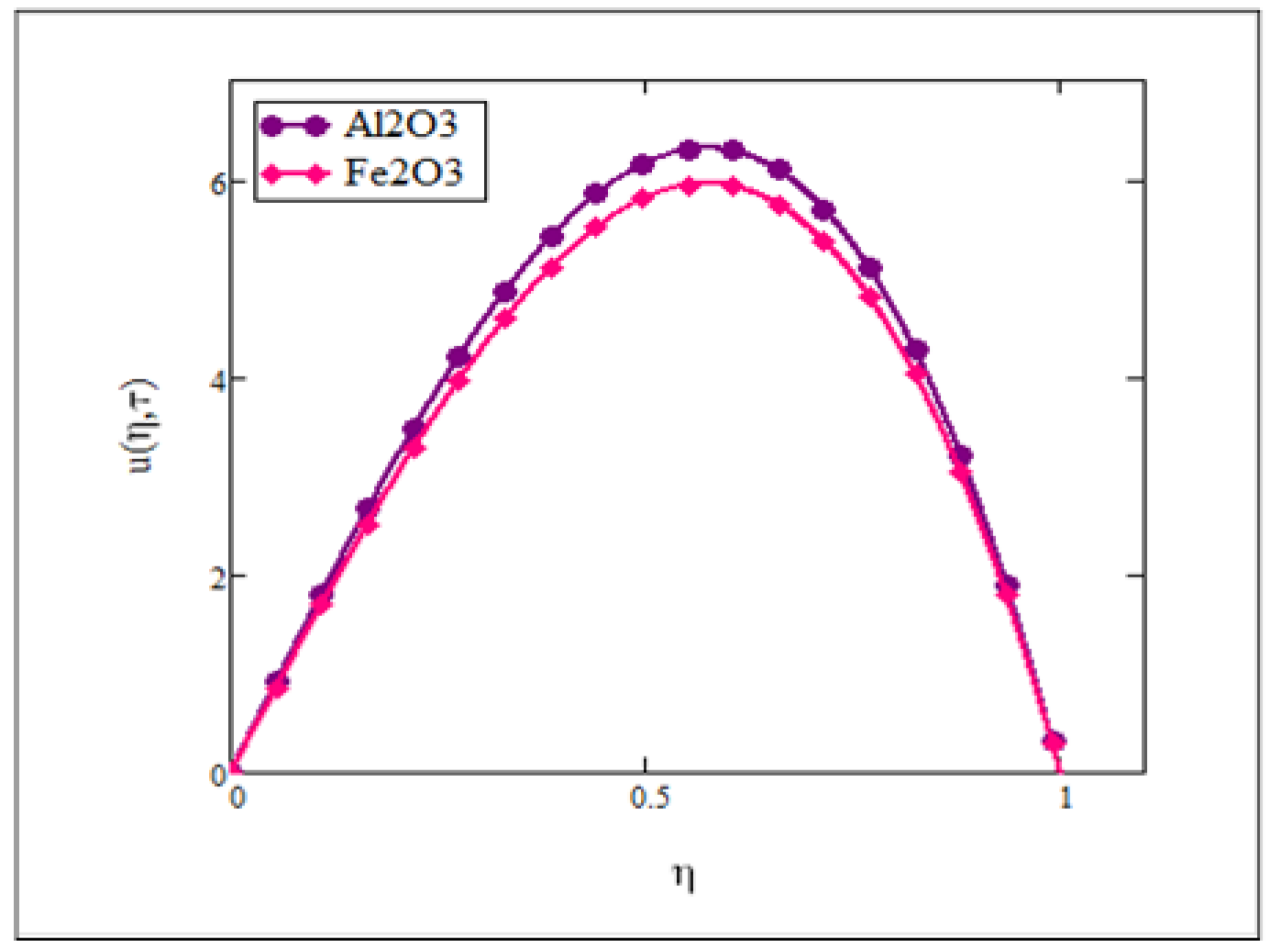
Comparison of ferrofluid (*Fe*_3_ *O*_4_) velocity with nanofluid (*Al*_2_ *O*_3_) velocity when *Gr* = 0.1, *φ* = 0.04, Re = 1, *Pe* = 1, *M* = 1, *λ* = 1, *τ* = 5 and *ω* = 0.2.

**Fig 6:**
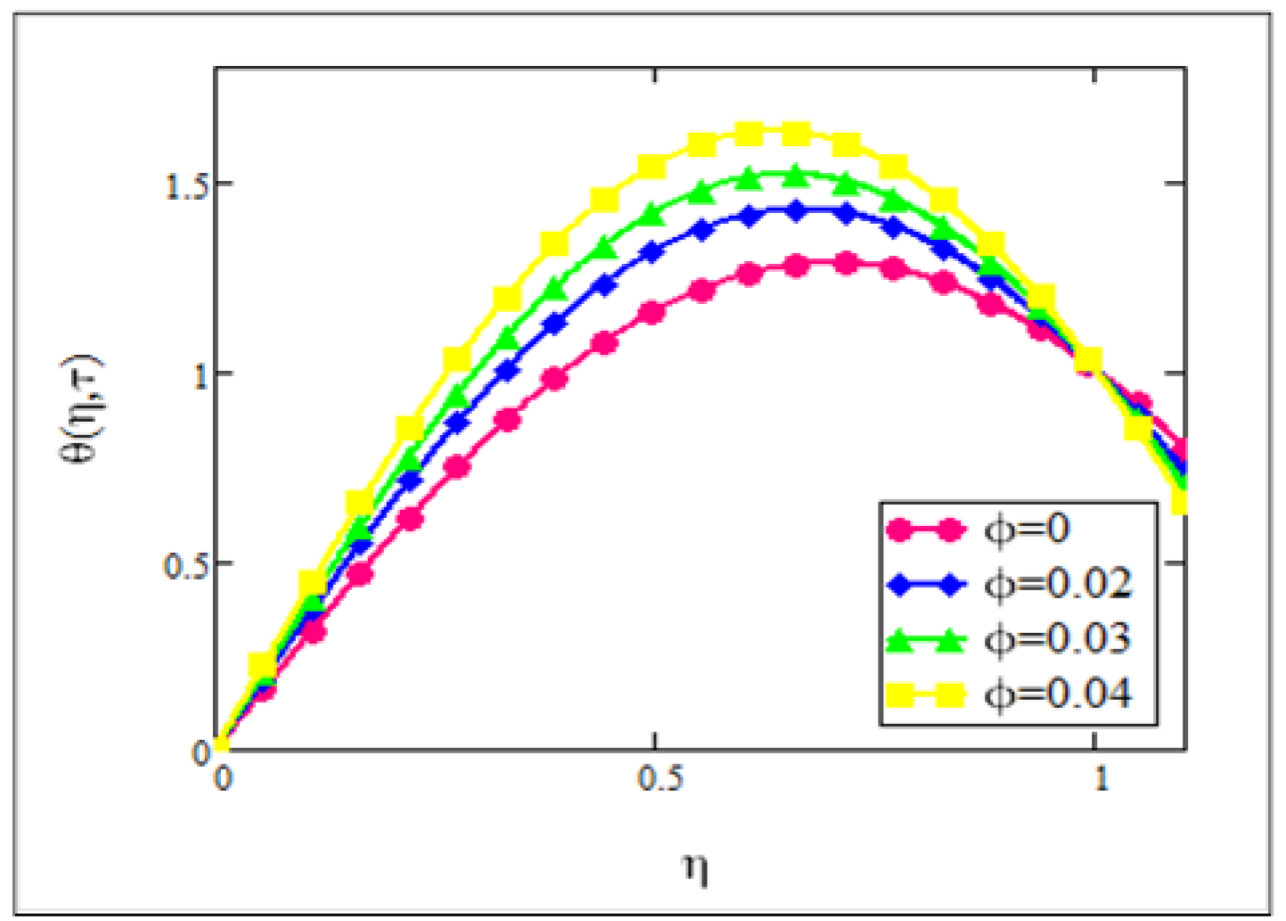
Temperature graph for *φ* when *N* = 1.5 and *τ* = 0.5.

**Fig 7:**
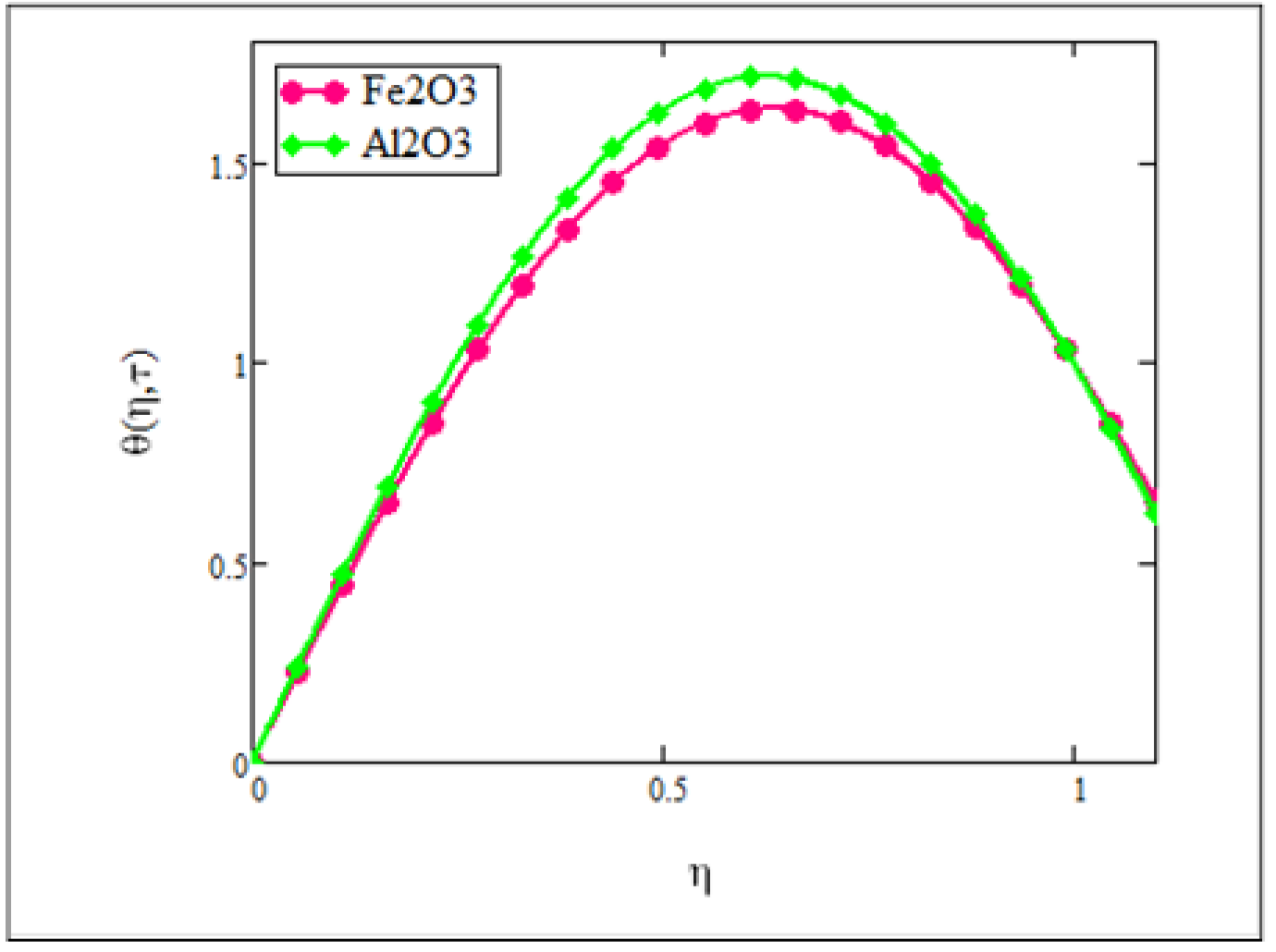
Comparison of ferrofluid (*Fe*_3_ *O*_4_) temperature with nanofluid (*Al*_2_ *O*_3_) temperature when *φ* = 0.04, *N* = 1.5 and *τ* = 0.5.

**Fig 8:**
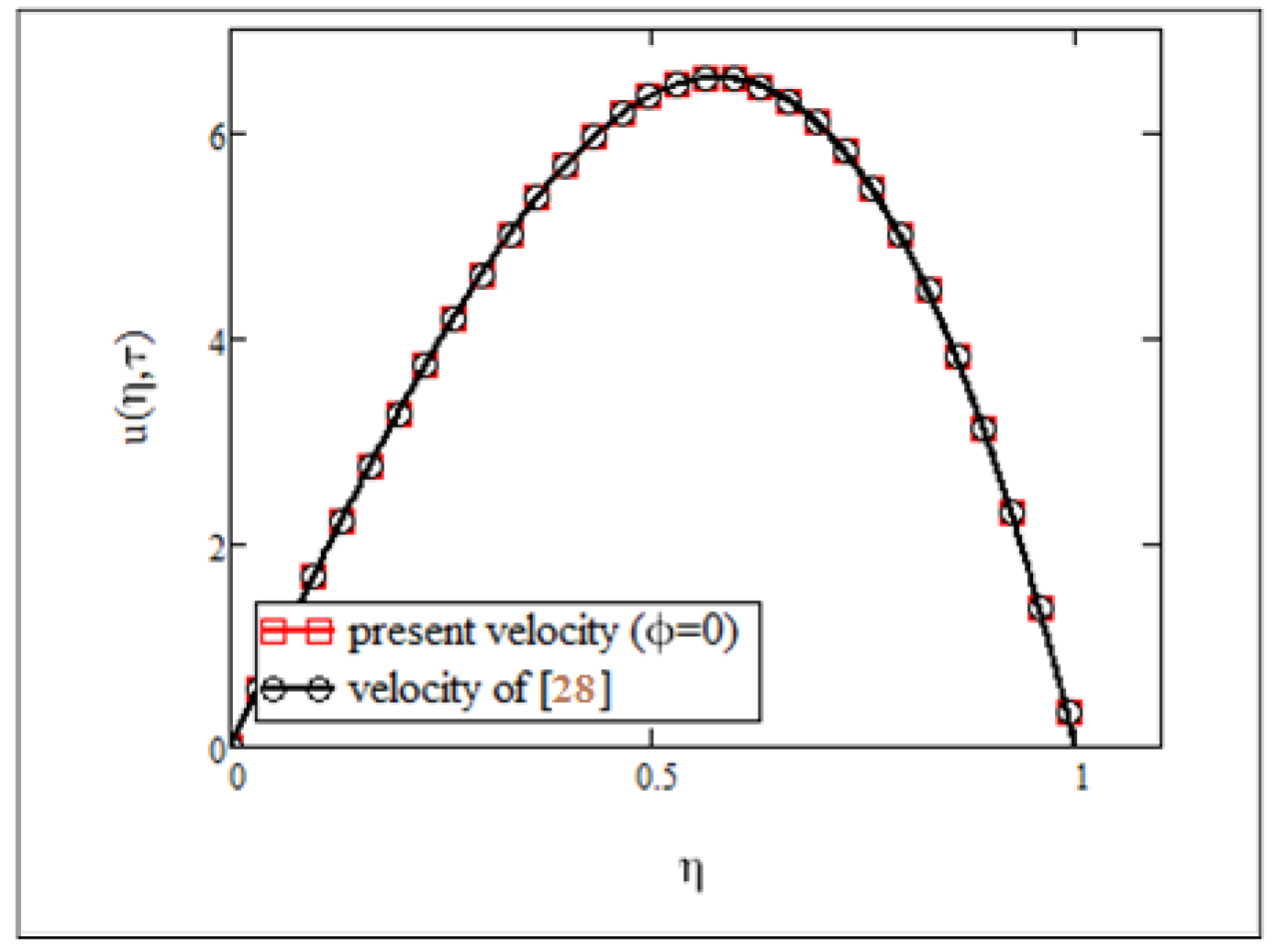
Comparison of present velocity results *φ* = 0 with published results of [30].

**Fig 9:**
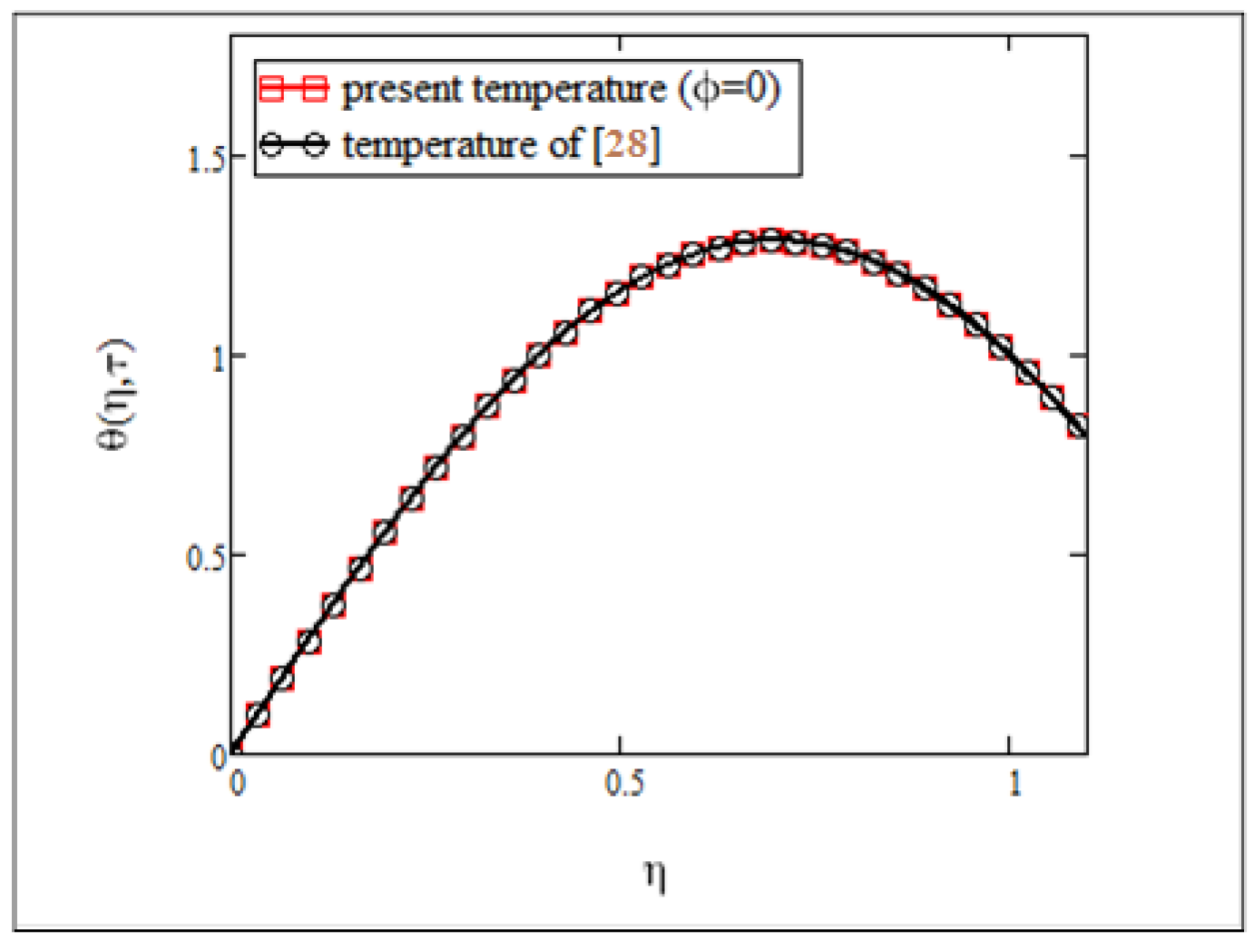
Comparison of present temperature results *φ* = 0 with published results of [30].

Fig 1 shows geometry of the problem whereas the analytical results were plotted in Figs 2–9. Parameters incorporated in the problem were the volume fraction of the nanoparticles *φ*, the magnetic parameter *M*, and the radiation parameter *N*. As mentioned in [14,15,22,26], the volume fraction of the nanoparticles was taken in the range of 0 ≤ *φ* ≤ 0.04. Note that volume fraction of the nanoparticles could not exceed 8%, as sedimentation takes place in this range.

Effect of *φ* on the flow of ferrofluid is studied in Fig 2. It was found that the velocity of the ferrofluid decreases with the increase of the volume fraction of the nanoparticles. Physically, it is true because when the volume fraction of nanoparticles increases in the base fluid, the conductivity and viscosity of the nanofluid increases in accordance with the theoretical model as predicted by Brinkman [28] for viscosity and classical effective thermal conductivity model, known as the Maxwell model [1]. Brinkman included particle volume concentration of up to 4% (roughly) and extended the Einstein model to *μ*_*nf*_ = *μ*_*f*_ / (1 − *φ*)^2.5^ and the Maxwell [1] model to *k*_*nf*_ = [*k*_*f*_(*k*_*s*_ + 2*k*_*f*_) − 2*φ*(*k*_*f*_ − *k*_*s*_)] / [(*k*_*s*_ − 2*k*_*f*_) + *φ*(*k*_*f*_ − *k*_*s*_)] Therefore, the velocity of ferrofluid is decreased with an increase in volume fraction because of the increase in viscosity.

Influence of *N* on the velocity is illustrated in Fig 3. Clearly, an increase in the radiation parameter contributes to the increase in velocity of the ferrofluid, which physically means that the fluid is emitting more heat. The increasing radiation parameter more specifically, increases the energy transfer rate to the fluid. Moreover, it can also be observed from this figure that with the increase in the radiation parameter, the viscosity of the ferrofluid was decreased which resulted in a increase in its velocity.

The velocity profile for different values of the magnetic parameter *M* is presented in Fig 4. It was observed that an increase in the magnetic parameter *M* led to a decrease in the velocity of ferrofluid. This trend was maintained less effectively with a further separation from the plate. Physically, this corresponds to the fact that effect of a transverse magnetic field on the electrically conducting fluid gives rise to a resistive type of force known as the Lorentz force which is similar to a drag force and upon increasing the value of *M*, the drag force increases and causes the fluid to move slowly. Increasing the magnetic parameter also increases the viscosity of the ferrofluid. Compared to other types of nanofluids, ferrofluid is highly affected by magnetic fields. Therefore, ferrofluid is also frequently referred as magnetic fluid. As we can see from this figure when the influence of the magnetic field was taken as zero (*M* = 0), the velocity was found to be greater than in the presence of the magnetic parameter. Fig 5 shows a comparison of velocities when two different types of nanoparticles namely, magnetite (*Fe*_3_ *O*_4_) and non-magnetic (*Al*_2_ *O*_3_) were added to the conventional base fluid. It was found that the velocity of the *Al*_2_ *O*_3_ nanofluid was greater than the velocity of the ferrofluid (*Fe*_3_ *O*_4_ nanofluid) when the volume fraction was *φ* = 0.04. This shows that the viscosity and thermal conductivity of the ferrofluid was greater than the *Al*_2_ *O*_3_ nanofluid.

Fig 6 shows the temperature distribution for different volume fractions. It is clear from this figure that with the increase of volume fraction, the temperature of the ferrofluid increases. Results from this study concurred well with the experimental results of Colla [11]. According to Colla [11], increasing the particle concentration means increasing the volume fraction which results in an increase in the thermal conductivity of the ferrofluid and therefore causes the temperature to increase. This means that an increase in volume fraction increases thermal conductivity while the viscosity of the ferrofluid decreases.

Fig 7 is plotted for the ferrofluid (when the added nanoparticles are of *Fe*_3_ *O*_4_) and the nanofluid (when the added nanoparticles are of *Al*_2_ *O*_3_) when the volume fraction is *φ* = 0.04. We found that the temperature was varied in the same way as we noticed in the case of velocity i.e. the temperature of the ferrofluid was smaller than the temperature of the nanofluid. The thermal conductivity and viscosity of the ferrofluid is temperature dependent. Thermal conductivity increases with increase in temperature whilst viscosity decreases with an increase in temperature. Furthermore, the viscosity of *Fe*_3_ *O*_4_ was greater than *Al*_2_ *O*_3_. This graphical illustration was found to be identical with the experimental results by Colla [11]. Finally, in Figs 8 and 9, the results of the present study were compared with those from Makinde and Mhone [30] in a non-porous medium. It can clearly be seen that when the volume fraction was *φ* = 0, results for velocity and temperature of the ferrofluid in this study were identical with the results obtained from regular fluids in [30].

## Conclusion

The present study examined analytically heat transfer in mixed convection flow of a ferrofluid inside a vertical channel. Analytical solutions are obtained using perturbation method. In order to make the ferrofluid, when the base fluid was electrically conducting under the influence of an external magnetic field, nanoparticles of magnetite (*Fe*_3_ *O*_4_) was added to the conventional base fluid which was water. Nanoparticles of different geometries can be used and therefore spherical shaped nanoparticles were chosen for this study. In order to enhance understanding results of the ferrofluid was compared with those of the nanofluid when the nanoparticles used were iron oxide (*Al*_2_ *O*_3_). Effects of some important parameters including radiation parameter, magnetic parameter and volume fraction of nanoparticles have been studied on thermal conductivity and viscosity of nanofluids. Two important thermophysical properties of nanofluids namely thermal conductivity and viscosity together with magnetic parameter were investigated. In this study it was found that these two properties affect the velocity and temperature of the ferrofluids. The viscosity and thermal conductivity of the spherical ferrofluids were increased with an increase in the volume fraction of nanoparticles. In contrast, viscosity of the ferrofluids was decreased with an increase in temperature. A comparison for both velocity and temperature between magnetic (*Fe*_3_ *O*_4_) and non-magnetic (*Al*_2_ *O*_3_) nanoparticles were also conducted. The results were found to be identical with the results predicted experimentally. Finally, this study showed that in the absence of the volume fraction of nanoparticles, the results of the present study were identical with published results.
